# Gene expression profiling as a prognostic tool in multiple myeloma

**DOI:** 10.20517/cdr.2021.83

**Published:** 2021-12-01

**Authors:** Harmony Black, Siobhan Glavey

**Affiliations:** ^1^Department of Haematology, Beaumont Hospital, Dublin D09 V2N0, Ireland.; ^2^Department of Pathology, Royal College of Surgeons in Ireland, Dublin D02 YN77, Ireland.

**Keywords:** Multiple myeloma, gene expression profiling, prognostication, risk stratification, risk-adapted therapies, SKY92, drug resistance

## Abstract

Multiple myeloma (MM) is an aggressive plasma cell malignancy with high degrees of variability in outcome, some patients experience long remissions, whilst others survive less than two years from diagnosis. Therapy refractoriness and relapse remain challenges in MM management, and there is a need for improved prognostication and targeted therapies to improve overall survival (OS). The past decade has seen a surge in gene expression profiling (GEP) studies which have elucidated the molecular landscape of MM and led to the identification of novel gene signatures that predict OS and outperform current clinical predictors. In this review, we discuss the limitations of current prognostic tools and the emerging role of GEP in diagnostics and in the development of personalised medicine approaches to combat drug resistance.

## INTRODUCTION

Multiple myeloma (MM) is an aggressive cancer of terminally differentiated B cells that arises within the bone marrow and typically manifests with hypercalcaemia, renal failure, anaemia, and osteolytic bone lesions (CRAB features)^[1]^. Almost all cases of MM evolve from the pre-malignant and asymptomatic monoclonal gammopathy of undetermined significance (MGUS) at a rate of 1% per year. However, in certain cases, the progression of MGUS to MM may be interceded by the more advanced asymptomatic disease, smouldering multiple myeloma, which evolves at a rate of 10% per year^[2]^.

Outcomes for MM patients have significantly improved in recent years, concurrent with the introduction of novel therapies, including immunomodulatory agents and proteasome inhibitors (PIs)^[3]^. However, improvements have not been consistent; some patients experience long remissions whilst others are faced with a dismal prognosis and survival of less than 2 years. These disparities are most likely due to genetic heterogeneity and clonal evolution along the disease course. Reflecting this inherent molecular complexity, MM is now recognised as an umbrella term for a collection of similar but genetically distinct cancers^[4]^.

Next-generation sequencing (NGS) methods, including RNA sequencing and gene expression profiling (GEP), have been instrumental in defining the molecular landscape of MM and in the past decade, many genes and pathways implicated in myelomagenesis and therapy resistance have been elucidated^[5,6]^. The expression data from such sequencing projects holds enormous potential and can be further mined to identify molecularly-distinct subgroups of patients and develop risk-associated gene signatures. Although several groups have published MM gene signatures, information on their comparative clinical utility is limited, and there is little consensus on their integration into routine diagnostic workup^[7-9]^.

Current methods of MM prognostication are based on cytogenetic analysis, which does not account for molecular complexity and therefore is not comprehensive enough to accurately discriminate between risk groups^[10]^. GEP is more sensitive and better informs risk-stratification^[11]^. Therefore, in this mini-review, we will compare current methods of MM prognostication to the best-performing gene signature presently available and a novel RNA-sequencing based classifier, their comparative prognostic performance, and we will examine the potential for developing risk-adapted personalised medicine approaches for MM.

## CURRENT METHODS OF PROGNOSTICATION

### Prognostication by cytogenetic profile

Almost all MM patients present with cytogenetic abnormalities, and indeed, it is a defining characteristic of this malignancy affecting clinical manifestations of the disease, response to treatment and prognosis^[12]^. Cytogenetic aberrations can broadly be classified into two groups. Primary events are believed to trigger founding MGUS clones, and secondary events that arise later in the disease course of which multiple can exist in any one patient^[10-13]^.

Primary events determine the disease classification and constitute two distinct subtypes of MM, hyperdiploid (HD) and non-hyperdiploid (NHD). HD patients present with a trisomy of one or more odd-numbered chromosomes, including 3, 5, 7, 9, 11, 15, and 17, and this group accounts for approximately 45% of all MM cases^[10,12,13]^. Around 55% of MM cases are NHD with reciprocal translocations involving the immunoglobulin (Ig) heavy chain gene on chromosome 14 and recurrent partner oncogenes. Rearrangements include t(4;14), t(6;14), t(11;14), t(14;16) and t(14;20), each of which causes deregulation of the *FGFR3-MMSET*, *CCND3*, *CCND1*, *MAF* and *MAFB* genes, respectively^[10,12,13]^. Deletion of chromosome 13 is also considered a primary event by the international myeloma working group (IMWG) and affects approximately 50% of patients^[14]^.

Secondary genetic events include deletion of chromosome 17p, deletion of chromosome 1p and gain or duplication of chromosome 1q21^[10,12,13]^. These events arise late in the disease course and virtually never in MGUS patients indicating their involvement in MM progression. Another recurrent secondary event is the translocation of the MYC oncogene located on chromosome 8q24, which is present in around 21% of cases^[10,12,13]^.

A patient’s risk status can be determined on the basis of their cytogenetic profile with high-risk (HR) generally accepted as having an overall survival (OS) of less than 2 years compared to standard-risk (SR), which carries an OS between 5-10 years^[10,12,13]^. An HR prognosis is diagnosed when at least one of the following are present: t(4;14), t(14;16) and t(14;20), deletion 17p and gain of 1q, detected by fluorescence *in-situ* hybridisation (FISH). An HR diagnosis can also be made if the patient has an NHD karyotype or deletion of chromosome 13. SR MM is diagnosed when a patient presents with any other cytogenetic abnormalities including t(11;14) and t(6;14)^[10,12,13]^.

#### The revised international staging system and its limitations

In 2005, the IMWG reported a novel, simple classifier, the international staging system (ISS), that could be used by clinicians to prognosticate patients into three groups based on serum β_2_-microglobulin and serum albumin levels^[14,15]^. Patients in ISS-I had a median OS of 62 months, ISS-II had a median OS of 44 months, and ISS-III had a median OS of 29 months^[15]^. A revised version of this classifier [revised international staging system (R-ISS)] was published in 2015 that incorporated cytogenetic profile and lactate dehydrogenase levels, as these were shown to have an independent prognostic value^[16]^. The R-ISS algorithm remains in use today as the sole prognosticator used by clinicians even though it fails to predict treatment response for each prognostic group and it does not inform clinical management of the disease, thus highlighting a clear need for improved prognostic biomarkers in MM.

### Prognostication by gene expression profiling

NGS evolution has permitted more extensive interrogations of the MM genome, and in recent years, numerous high-throughput GEP studies have been instrumental in the identification of distinct molecular subgroups which are significantly associated with a patient’s risk and survival^[6,7]^. These subgroups are denoted by specific gene signatures, of which more than 20 are published^[11]^, including *UAMS-17*, *UAMS-70*, *UAMS-80*, *IFM-15*, *MRC-IX-6*, and *MILLENNIUM-100*^[17-22]^. These signatures have been reviewed elsewhere and are beyond the scope of this mini-review^[7,8]^. This review will elaborate on the Erasmus Medical Center 92 gene signature (*EMC-92*), also referred to as *SKY92*, demonstrated to be the best prognostic tool currently available for MM^[23]^, and we will also discuss a novel RNA-sequencing based signature comprised of only five genes, published by Zamani-Ahmadmahmud *et al.*^[24]^ in 2021.

#### The SKY92 signature

In 2012, Kuiper *et al*.^[23]^ published a novel gene signature, *SKY92* (also known as *EMC92*), that predicts survival and encompasses the underlying biology of HR patients. The HOVON-65/GMMG-HD4 clinical trial recruited newly diagnosed (ND), transplant eligible MM patients and Kuiper *et al*.^[23]^ used the gene expression microarray data from this trial as their training dataset. They isolated their signature by first filtering the raw microarray data for probe set intensity followed by univariate Cox regression analysis of 27,680 probe sets. This yielded 1093 probes associated with progression-free survival^[23]^. Subsequently, supervised principal-components-analysis (PCA) with simulated annealing led to the identification of a 92-probe set signature, termed SKY92. For each gene in the classifier, there is a corresponding weighting coefficient which should be multiplied by the normalised gene expression for the probe, and these values should be summed for all 92 genes to produce a final signature score. An EMC92 signature score of greater than or equal to 0.827 classifies a patient as HR with an OS of less than 2 years, and patients with a score less than 0.827 are SR^[23]^.

The group performed signature validation in four independent datasets; UAMS-TT2, UAMS-TT3, APEX, and MRC-IX. For the UAMS-TT2 cohort, 19.4% of patients were classified as HR by EMC92 signature, 16.2% of UAMS-TT3 were HR, and 20.2% of MRC-IX were HR^[23]^. They demonstrated that EMC92 could identify HR in both transplant eligible and ineligible groups comprising the MRC-IX cohort, with 23.9% of ineligible patients classified as HR compared to 16.8% of transplant eligible patients^[23]^. Moreover, the APEX dataset contains relapsed MM patients, of which 16.3% were HR by EMC92, proving the signature is not limited to ND groups^[23]^. Therefore, the authors were able to demonstrate that their signature accurately, and reproducibly identifies HR patients who have significantly reduced OS compared to the SR groups.

Kuiper *et al*.^[23]^ performed additional multivariate analysis of the HOVON-65 data and found that although del (17p) and ISS criteria were independently prognostic for MM, their gene signature was the best performing prognosticator across all trialled datasets, independent of all other classifiers. Moreover, when assessed in comparison to existing signatures including UAMS-17, UAMS-70, UAMS-80, IFM-15, GPI-50, MRC-IX-6, and MILLENIUM-100, the group found that EMC-92 was superior in its prediction of OS, robustly identifying truly HR populations^[23]^. A subsequent study from the Erasmus group demonstrated that when EMC92 and ISS criteria are used in combination to determine risk, four distinct groups of patients can be identified, low risk (LR), intermediate-low (IL), HR, and intermediate-high^[25]^. This EMC92-ISS classifier is the single best performing tool for prognostication currently available, accurately identifying four groups with OS ranging from 24 months for the HR patients to 61 months for IL, and median OS was not reached after 96 months for the LR group^[23,25]^. 

#### SKY92 predicts treatment response

Improving prognostication at the time of diagnosis could lead to augmented treatment protocols, either escalating or de-escalating therapy based on a patient’s risk category. Recently, preliminary data from the PROMMIS trial (NCT02911571) was reported by Biran *et al*.^[26]^, which explored the impact of MMprofiler™ (SKY92) scores on treatment intentions in ND MM patients. Of the 147 patients tested, MMprofiler™ results re-classified 16 cases that were SR by FISH, as HR according to SKY92, and this led to clinicians escalating treatment in 15 of these cases. Interestingly, 50% of the patient cohort were classed as HR by FISH at diagnosis, and following SKY92 testing, 46 of these patients were determined to be SR^[26]^. This caused clinicians to de-escalate treatment for 31 of the patients. Significantly, MMprofiler™ results led to a change in treatment course in 37% of the patients enrolled in the study, highlighting the value of this assay for clinicians in tailoring treatment to each patient’s disease^[26]^.

The HOVON-87/NMSG-18 trial enrols elderly MM patients and investigates the efficacy of melphalan-prednisone-thalidomide followed by thalidomide maintenance (MPT-T) with melphalan-prednisone-lenalidomide followed by lenalidomide maintenance (MPR-R)^[27]^. A 2020 publication from Kuiper *et al*.^[28]^ analysed the available R-ISS and SKY92 data for 168 of these patients to determine if MMprofiler™ results impacted response to therapy. In this cohort, they distinguished three distinct groups with significantly different survival, SKY-RISS I (SR, 3-year OS 88%), SKY-RISS II (all others, 3-year OS 66%), and SKY-RISS III (HR, 3-year OS 26%)^[28]^. Their analysis showed that HR patients in this cohort responded better to the MPR-R regimen than the MPT-T regimen, with median OS of 55 and 14 months, respectively^[28]^. This study validates not only the prognostic benefit of GEP testing by SKY92 but its predictive value. Although further validation across additional clinical trial datasets is required, in the future, the results of a patient’s GEP or SKY92 test could inform their treatment protocol.

#### A 5-gene RNA-sequencing based prognostic signature

A recent 2021 publication from Zamani-Ahmadmahmud* et al*.^[24]^ described a novel gene signature comprised of only 5 genes and identified using publicly available RNA-sequencing datasets. The authors chose to mine RNA sequencing datasets as they have previously been described in the literature to hold more accurate gene expression data than microarray repositories^[24,29]^. Using the MMRF-CoMMpass resource^[30]^, the group employed a stringent four-filter analysis pipeline, first performing univariate cox proportional hazard analysis to identify only genes significantly associated with OS (*P *< 0.001). Their second filter utilised six different class prediction algorithms, which identified genes predicting for long-and short-time survival (≥ 2 years and ≤ 2 years, respectively), this gene list was then validated in the GSE24080 dataset. Finally, the refined gene list underwent multivariate Cox proportional hazards analysis in their training dataset to select genes significantly associated with OS (*P *< 0.001 and false discovery rate < 5%)^[24]^. Their final signature was externally validated in four further datasets (GSE2658, GSE6477, GSE136324, and GSE57317)^[24]^. A risk score is calculated by multiplying the normalised expression value of each gene in the signature by their corresponding regression coefficient. A threshold value of the 75th percentile is applied to the calculated risk scores to stratify patients into LR and HR groups^[24]^.

The resulting signature is comprised of five genes, *CCT2*, *PRKDC*, *NONO*, *UBE2A*, and *CKS1B*, and in the training and validation datasets, it accurately discriminated HR and LR groups (*P* < 0.001)^[24]^. Furthermore, in external datasets, the signature predicted for OS in MM patients with 3-year OS rates of 86% and 52% for LR and HR groups in GSE24080, and 94% and 36% for LR and HR groups in GSE357317^[24]^. Multivariate Cox analysis indicated that their signature was independently prognostic for MM OS, outperforming R-ISS criteria^[24]^. In comparison with other available gene signatures, the authors observed a slight degree of overlap between genes and noted that many genes in prior studies did not correlate with OS in their RNA sequencing datasets; however, comparative prognostic performance between their signature and others was not examined^[24]^. Noteworthy, however, Zamani-Ahmadmahmud *et al.*^[24]^ performed pathway enrichment analysis of their gene list pre-filtering, which showed significant enrichment of genes in cell cycle, cell division and sister chromatid cohesion pathways. This is in direct concordance with SKY92 enrichment data from Kuiper *et al.*^[23]^, suggesting overlap between the signatures.

What remains to be discussed is whether a targeted capture sequencing approach for these five genes is possible within the clinical laboratory environment, and moreover, the issue of RNA sequencing data analysis is often a barrier for treating physicians and could prevent the introduction of this test in the clinic. In addition, Zamani-Ahmadmahmud *et al.*^[24]^ also discuss the issue of complexity and size of available gene signatures and the impracticality of utilising large gene sets in clinical diagnostics. Whilst this is in principle true, the issue of commercial utility and data analysis has been addressed by Skyline Dx, a biotech company spin-out from Rotterdam Erasmus Medical Centre. In collaboration with Kuiper *et al*.^[23]^, the developers of the EMC92 signature, they developed MMprofiler™, a CE-IVD microarray assay that tests a patient’s SKY92 profile and assigns them either an HR or SR score^[31]^. The analysis of the microarray data is performed by Skyline Dx, and a clear and concise report is returned to the physician, enabling them to make an informed decision on the treatment protocol.

As reported by van Beers *et al*.^[32]^ in 2020, the MMprofiler™ assay underwent successful analytical validation. Intra and inter-run precision was tested for five SKY92 HR, and two SR specimens across 18 runs and all independent variables were tested in duplicate. Repeatability was shown to be high, not exceeding the maximum allowed standard deviation (0.45) for any specimen tested^[32]^. In addition, the analytical variance was assessed across three independent laboratories for eight HR, and four SR reference samples and reproducibility was found to be high with > 77.8% experimental concordance between laboratories^[32]^. For primary bone marrow samples tested, the authors report 100% concordance between expected and observed results for assay stability, reproducibility and repeatability^[32]^. As demonstrated, this assay pipeline is accessible, standardised and achievable across hospital laboratories and therefore of high clinical value for MM patients. Therefore, the clinical utility of larger gene signatures cannot be readily dismissed.

## OVERCOMING DRUG RESISTANCE AND MOVING TOWARDS PERSONALISED MEDICINE APPROACHES IN MULTIPLE MYELOMA

The past decade has seen a drastic increase in the arsenal of drugs available to treat MM, with PIs and immunomodulatory drugs becoming mainstays of front-line therapy^[32]^. Contemporaneous with this, survival has significantly improved, and the duration of response has increased^[32,33]^. Nevertheless, MM remains incurable in 2021 and disease relapse and therapy refractoriness are persistent challenges in the clinical management of the disease. NGS studies have shed light on resistance mechanisms in MM with mutations in KRAS, NRAS, TP53 and DIS3 shown to affect response to therapy and epigenetic modifications which alter tumour suppressor expression are also known contributors^[34]^. Furthermore, the complex interaction of MM cells within the bone marrow microenvironment can affect the efficacy of chemotherapeutic drugs and secretion of soluble factors from bone marrow stromal cells can activate signal transduction pathways such as JAK-STAT, which ultimately drives therapy resistance^[35,36]^.

Harding *et al*.^[37]^ published an outstanding review on the subject of therapy resistance in MM in 2019. Their comprehensive article discusses the numerous mechanisms by which MM cells overcome not only standard of care drugs but novel targeted therapies. Harding *et al*.^[37]^ highlight the requirement for timely integration of prospective disease profiling in the clinic to identify known biomarkers of resistance, thereby enabling patients to be matched to a more appropriate treatment plan from the outset. Coupling molecular risk stratification with data-driven treatment selection customised to each patient will undoubtedly lead to improved responses and prolonged survival, and thus, efforts should focus on their urgent translation to the clinic.

For HR patients, outcomes are especially poor, and in particular, they would benefit from the introduction of novel targeted therapies, which are lacking in MM^[38]^. One such targeted treatment was reported by Kumar *et al*.^[39]^ in 2016 they presented the results of a phase I open-label study of Venetoclax, a BCL-2 inhibitor, to treat relapsed/refractory MM. BCL-2 is a protein essential to the cell death response and is highly expressed in patients harbouring t(11;14) abnormalities^[40]^. Their study showed a promising 21% overall response rate, and 15% of the 66 patients enrolled achieved a very good partial response or better^[39]^. Though not yet FDA-approved for the treatment of MM, the potential of Venetoclax as a salvage therapy for relapsed refractory patients is promising and similar advancements are needed urgently.

Given the evident clinical utility of signatures such as EMC92, it is possible that the genes within these prognostic signatures could represent novel predictive biomarkers, and there is significant untapped potential for their therapeutic targeting. By identifying commonalities in the pathways and processes related to the genes in these signatures through pathway enrichment analysis, new druggable targets can be unearthed. Kuiper *et al*.^[23]^ performed ingenuity pathway analysis of the *SKY92* genes and reported enrichment of cell cycle, DNA replication and DNA repair pathways. Altered function of these pathways is certainly a driving force in SKY92-defined HR MM and may also represent novel mechanisms of therapy resistance in this poor-outcome group. Therefore, future studies should aim to elucidate the relationship between HR MM defined by gene signatures such as *SKY92* and response to standard of care therapies.

In recent years, efforts have been made to develop molecular predictors of drug resistance in MM, which could direct clinical management of the disease. In a 2017 publication, Mitra *et al.*^[41]^ performed chemosensitivity assays with PIs bortezomib, carfilzomib, ixazomib, and oprozomib in a panel of MM cell lines. Subsequent transcriptomic sequencing data for treated cells were analysed via machine learning algorithms yielding a novel 42 gene signature that was able to distinguish between the good and poor response to PIs as well as outcomes in publicly available datasets^[41]^. In a similar vein, a more recent 2020 paper from de Boussac *et al*.^[42]^ described the analysis of GEP data from three studies (UAMS-TT2, UAMS-TT3 and the Heidelberg-Montpellier cohort) to determine the expression profiles of 661 known kinase-related genes in MM and their association with survival. Interestingly, 36 kinase genes were shown to be significantly associated with OS across all three cohorts, allowing the authors to build a novel GEP signature based on the MM kinome^[42]^. Therapeutic targeting of seven genes in this signature is possible with kinase inhibitors as demonstrated in MM cell lines as single agents and in combination with standard of care therapies. This study clearly demonstrates the potential for kinome-related risk stratification and molecular therapeutic targeting on kinome-HR patients^[42]^. Moreover, it should be noted that de Boussac *et al*.^[42]^’s signature is enriched for kinases involved in cell cycle and mitotic pathways, thus showing marked concordance with the *SKY92* gene signature.

A final relevant study carried out by Herviou *et al.*^[43]^ described a significant upregulation of polycomb repressive complex 2 (PRC2) genes (*EZH2*, *SUZ12* and *EED*) in MM primary samples and cell lines compared to normal bone marrow controls, all of which are associated with poor OS. The group assessed the potential for therapeutic targeting of PRC2 genes via chemosensitivity assays with EZH2 inhibitor EPZ-6438, which inhibited cell proliferation and induced apoptosis-mediated cell death. Moreover, these effects were potentiated in combination with lenalidomide^[43]^. The authors present a novel prognostic GEP signature based on 15 genes associated with EZH2 inhibition (EZ Score). Higher EZ signature scores predict worse OS and identify a subset of patients who may benefit from *PRC2* gene inhibitors, enabling reactivation of tumour suppressors and transcription factors^[43]^.

Although a ways in the future, it would be optimal to screen MM patients at diagnosis for their gene expression profile and based on their individual signature, match them to a combination of targeted treatments and standard of care drugs unique to their disease with the aim of inducing superior outcomes, longer remissions, and potentially cure. A truly exciting prospect, progressing towards a personalised treatment protocol for each patient based on molecular subgroup should be the goal for MM researchers and clinicians.

## REMAINING CHALLENGES AND FUTURE DIRECTIONS

The clinical utility of GEP assays to improve risk-stratification and patient outcomes is evident; however, the lack of inter-laboratory consensus on the use of one standardised signature and its integration into routine diagnostic workup is the major barrier to their use. Moreover, the prospect of analysing complex sequencing data for each patient tested by GEP can be a challenge for physicians and may further hinder the use of these tests in the clinic. It must also be considered that with several signatures available, each comprised of different and rarely-overlapping genes, would it serve patients better to prognosticate based on a combination of signatures.

Indeed, this question was a focus of Chng *et al.*^[8]^’s 2016 study, which explored the prognostic utility of MM gene signature combinations. The authors selected nine published signatures significantly associated with MM OS in at least one of three publicly available datasets used in their analysis. All possible combinations of the nine signatures were tested, and survival analysis was performed for each individual combination using the log2 transformed normalised gene expression data from each of the 3 MM datasets^[8]^. The signatures and datasets used in this study are outlined in Table 1.

**Table 1:** Prognostic performance of gene signature combinations

**Dataset**	**Overall survival 9-signature combination *P*-value**	**Overall survival EMC92 + HZDCD *P*-value**
University of Arkansas Medical School GSE2658	2.17 × 10^-11^	3.79 × 10^-10^
APEX/SUMMIT/CREST GSE9782	7.18 × 10^-6^	4.39 × 10^-9^
HOVON-65/GMMG-HD4 GSE19784	6.63 × 10^-14^	2.22 × 10^-16^
**9 gene signatures included in combination**		
**UAMS80** 80-gene prognostic signature UAMS researchers	**IFM15** Intergroupe Francophone du Myelome 99 clinical trial	**EMC92/SKY92** Signature derived from HOVON-65/GMMG-HD4 clinical trial data
**UAMS70** 70-gene prognostic signature UAMS researchers	**HZDCD** Cell death signature	**CNTI** Centrosome index
**PI** Proliferation signature	**HMCL** 7-gene prognostic signature from MM cell line study	**CINGECS** Chromosome instability signature

A total of nine gene signatures were trialled in combination to test their joint prognostic ability in a 2016 study conducted by Chng *et al.*^[8]^. The combinations were trialled in three datasets; GSE2658, GSE9782 and GSE19784. All possible pairwise signature combinations were also trialled, and their performance was compared against single signatures and all signature combinations. EMC92 + HZDCD was identified as the single best combination with superior separation of standard-risk and high-risk groups within each patient cohort and a more significant *P*-value. Data referenced in this table is adapted from Chng *et al*.^[8]^.

Gene signature scores were aggregated by averaging their index values to yield a final combination score, and this was found to identify more significant *P*-values than the alternative PCA approaches. Their analysis showed that increasing the number of signatures in the combination improved the prognostic performance. However, they highlight one gene signature combination, EMC92 + HZDCD, that performs best across all tested datasets and prognosticates significantly better than either signature alone^[8]^. As such, this signature combination should be referred to as a benchmark to which the performance of future signatures should be compared. Moreover, Chng *et al*.^[8]^ propose that their combination approach can be easily integrated into analysis pipelines for GEP tests, and their script is available for all combinations or EMC92 + HZDCD alone. Although this study has highlighted the potential for routinely combining signatures, we must consider how feasible this is in reality as the EMC92 workflow is standardised, and all analysis is performed by Skyline Dx. Therefore, the willingness of clinical laboratory staff to implement a separate in-house analysis pipeline could pose a barrier. Moreover, the likelihood of two or more GEP tests being performed for each MM patient is small outside of a research environment, and so this could further limit the implementation of this combination approach.

It is clear that there now exists a myriad of relevant prognostic and predictive biomarkers for MM, each with their own value and applicability. How to select one signature to tests for, or to weigh up the clinical utility of one over the other remains to be seen as it is impracticable to incorporate all into routine clinical use, and indeed, not all would prove valuable enough to warrant this. Therefore, future efforts should focus on weighing up the cost-benefit relationships of existing predictors and determining their realistic usefulness in the clinical management of MM patients.

Adding a further layer of complexity to MM treatment is the issue of tumour spatial heterogeneity. An elegantly executed study by Rasche *et al.*^[44]^ highlighted the multi-region involvement of MM. Their study involved sequencing of paired tumour samples from 42 ND and 11 MM patients who had received prior treatment. One biopsy sample was aspirated from the iliac crest and one from another site guided by radiology results^[44]^. Remarkably, NGS data revealed genomic heterogeneity between the paired aspirates in most patients and in certain cases, the karyotypic profiles were not concordant between aspirate sites. Perhaps most interesting is that in 25% of the patients included in the study, their risk status determined by GEP (GEP70) was discordant between aspirate sites^[44]^. Subsequent analysis of a larger patient cohort (*n* = 263) for which paired NGS data was available revealed that GEP70 scores predicted for poor OS irrespective of sampling site with no significant difference in survival observed between patients HR by GEP at both sites versus patients HR at one site only^[44]^. Nevertheless, their results highlight the potential for truly-HR MM patients to be missed due to spatial heterogeneity of malignant clones and also underscores the necessity for multi-drug approaches, confirming that a one-size-fits-all approach to treatment is not sufficient in the management of this disease.

## CONCLUSION

**Figure 1:**
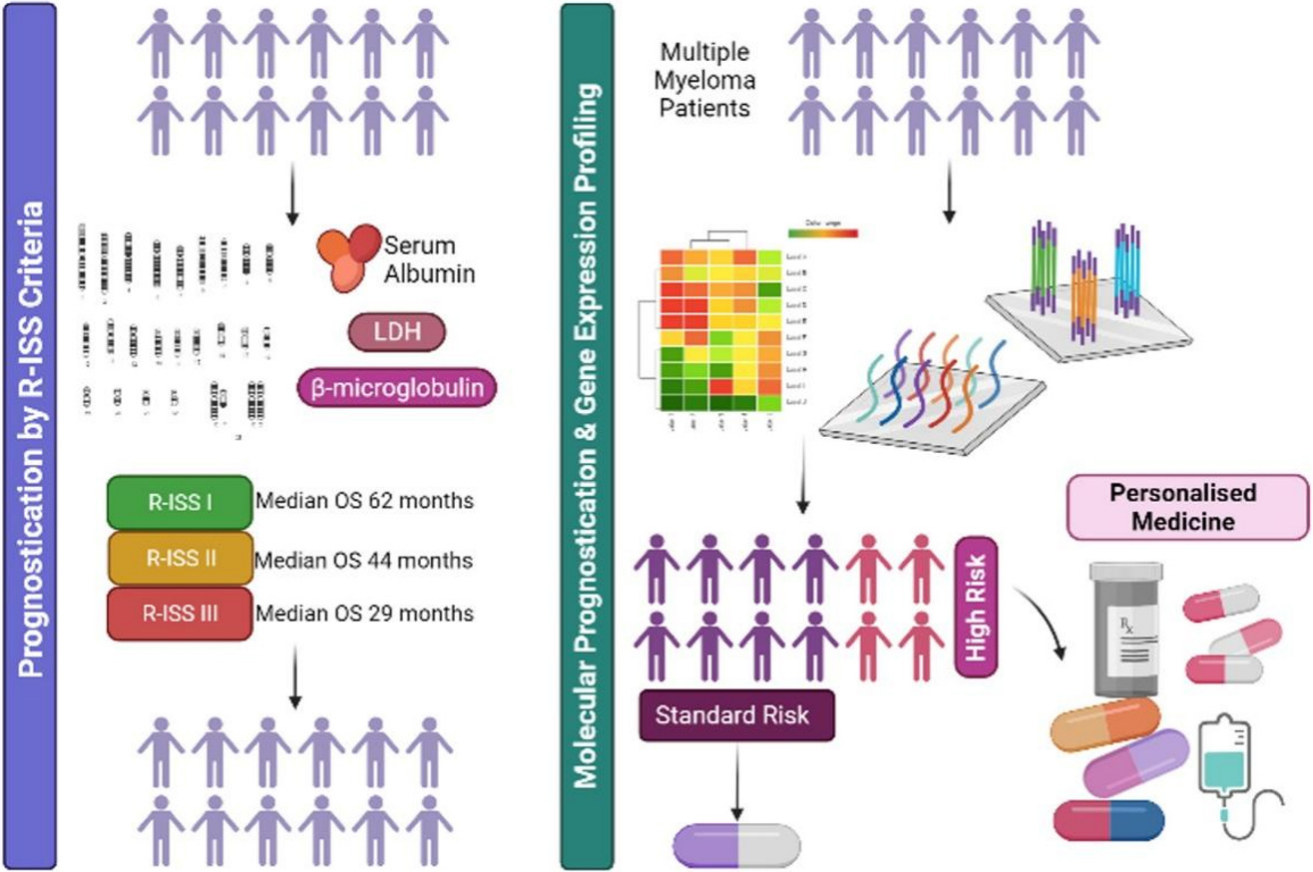
Presently, multiple myeloma patients are prognosticated based on R-ISS criteria which do not account for molecular complexity and thus cannot accurately discriminate between risk groups. Moreover, R-ISS categories do not inform treatment protocols. Molecular prognostication via gene expression profiling provides superior risk-stratification, accurately distinguishing truly high-risk (HR) patients from standard-risk (SR). This, in turn, can inform treatment protocols, directing SR patients towards less-intensive therapies and HR towards more personalised multi-drug approaches based on their individual genetic sensitivity profiles. Created with BioRender.com. R-ISS: Revised international staging system.

In conclusion, GEP is a sensitive and robust method of risk stratification for MM which outperforms current classification tools, including cytogenetic analysis by FISH and R-ISS criteria. As evidenced in recent clinical trial data, the SKY92 signature aids clinicians in disease classification and informs treatment decisions which can significantly impact patient outcomes and quality of life. Therefore, future studies should focus on the integration of commercial GEP assays into routine diagnostic workflows and the development of targeted, risk-adapted treatment protocols to drive improved outcomes for MM patients with particularly poor prognosis [Figure 1].

## DECLARATIONS

### Copyright

© The Author(s) 2021.
